# Estimation of the tumor size at cure threshold among adult patients with adrenocortical carcinoma: A populational-based study

**DOI:** 10.1016/j.heliyon.2024.e28160

**Published:** 2024-03-22

**Authors:** Yi Huang, Libo Liu, Qinghua Gan, Zefeng Shen, Yuhui Yao, Chengxiao Liao, Sihong Lu, Yitong zou, Yaqiang Huang, Jianqiu Kong, Xinxiang Fan

**Affiliations:** a Department of Urology, Sun Yat-Sen Memorial Hospital, Sun Yat-Sen University, Guangzhou, 510120, Guangdong, PR China; b Guangdong Provincial Key Laboratory of Malignant Tumor Epigenetics and Gene Regulation, Sun Yat-Sen Memorial Hospital, Sun Yat-Sen University, Guangzhou, 510120, Guangdong, PR China; c Guangdong Provincial Clinical Research Center for Urological Diseases, PR China; d Department of Urology, Zhongshan City People's Hospital, Sunwen East Road, Zhongshan, 528400, Guangdong, PR China

**Keywords:** Tumor size, Adrenocortical carcinoma, Chemotherapy, Prognosis

## Abstract

**Background:**

The prognostic significance of tumor size with adrenocortical carcinoma (ACC) patients has not yet been thoroughly evaluated. Our objective was to investigate the influence of tumor size on prognostic value in adult ACC patients.

**Methods:**

The Surveillance, Epidemiology and End Results Program (SEER) was employed to identify adult ACC patients who had been diagnosed from 2004 to 2015. The “*X*-Tile” program determined the optimal cutoff value of tumor size. Cancer-specific survival (CSS) and overall survive (OS) were estimated. The survival outcomes and risk factors were analyzed by the Kaplan-Meier methods and the multivariable cox regression respectively.

**Results:**

A total 426 adult ACC patients were included. Univariable and multivariable cox analysis revealed age, larger tumor size and metastasis as consistent predictors of lower CSS and OS. The optimal cutoff value of tumor size was identified as 8.5 cm using *X*-tile software, and Kaplan-Meier method showed dramatic prognostic difference between patients with larger tumors (＞8.5 cm) and smaller tumors (≤8.5 cm) (log-rank test, *P* < 0.001). Subgroup analyses revealed no statistical significance and a consistent proportionate effect of tumor size on CSS and OS across all eight pre-specified subgroups. Interestingly, an additional subgroup analysis showed that ACC patients could not benefit from chemotherapy in terms of CSS and OS.

**Conclusion:**

The study suggests that tumor size is a crucial prognostic factor in ACC patients and a cutoff value 8.5 cm might indicate a poor outcome. Given the limitations of the available data, it is challenging to conclusively determine the benefit of chemotherapy in adult ACC patients across different tumor size ranges.

## 1 Introduction

Adrenocortical carcinoma (ACC), a rare and extremely aggressive endocrine tumor that can affect both children and adults, is estimated incidence of 0.7–2.0 per million people each year [1]. ACC has a poor prognosis with 5-year survival rate ranging from 16% to 38% [2,3], and median survival time ranges from 14 months to 28 months [4]. Besides, due to high degree of malignancy and recurrence rate, two-thirds of ACC patients encountered recurrence within 2 years after surgery, including local recurrence and metastasis [5].

Currently, the treatment of ACC mainly includes surgery, radiotherapy and adjuvant chemotherapy. Entire resection of the carcinoma may be the only curative treatment for non-metastatic ACC [6]. The application of adjuvant therapy, such as the administration of mitotane, has the potential to enhance the prognosis of ACC patients, while there is a lack of convincing evidence to demonstrate whether adjuvant chemotherapy will decrease the recurrence rate and improve survival outcome [7]. Radiotherapy was seldom used in the treatment of ACC, because a small Pre-2000 series data showed radiotherapy neither improving any overall survival nor decreasing the recurrence of ACC [[8], [9], [10]]. However, Kan Wu et al. suggested that radiotherapy after radical surgery might be related to better prognosis in non-metastatic ACC patients [11]. Additionally, mitotane combined with IGF1R inhibitor cixutumumab (IMC-A12) treatment for metastatic ACC didn't improve survival outcomes and had potentially fatal toxic effect, while biological activity was demonstrated in some patients [12].

As the expectancy for ACC is dismal and the therapy is monotonous and uncertain, which is related to the tumor stage, age, tumor pathologic grade, hormone level, and molecular mechanisms [13]. The dimension of the tumor is an essential clinicopathological factor that influences the prognosis of many solid tumors [14], such as lung cancer [15], hepatocellular cancer [16] and gastric cancer [17]. In parathyroid carcinoma, tumor size has better prediction outcome than male gender, lymph node metastasis, and other factors [18]. However, tumor size of ACC remains controversial whether it can accurately predict the prognosis of the ACC and as a reference index exemption from adjuvant chemotherapy.

The genomic characteristics of ACC are different between pediatric and adult patients, and the prognosis of ACC are also different, which we have reported previously [19,20]. While the frequency of germline TP53 mutations in children diagnosed with ACC is notably high, ranging from 50% to 97%, it is significantly lower in adults, at around 5.8% [21]. This genetic predisposition in pediatric cases, particularly evident in Southern Brazil where the incidence of pediatric ACC is 15 times higher than in other regions, is a key differentiator [22]. Moreover, the clinical manifestations of ACC in children tend to be more severe, with worse histopathological features, yet interestingly, they often experience better clinical outcomes compared to adults [23]. In contrast, adults with ACC typically show a direct correlation between pathological findings and clinical prognosis.

Thus, the purpose of this study was to determine the impact of tumor size on prognostic value in adult ACC patients, as well as to determine the optimal cutoff value for tumor dimension to identify ACC patients with an unfavorable prognosis and to formulate precision treatment.

## 2 Materials and methods

### 2.1 Date source

The population of this study was obtained from the Surveillance, Epidemiology, and End Results Program (SEER). SEER*Stat software (SEER*Stat 8.4.01) was used to obtain related data. The research data was obtained in December 2022.The population was selected from SEER Research Plus Data, 17 registries, Nov 2021 Sub (2000–2019).

### 2.2 Data extraction

**Fig. 1:**
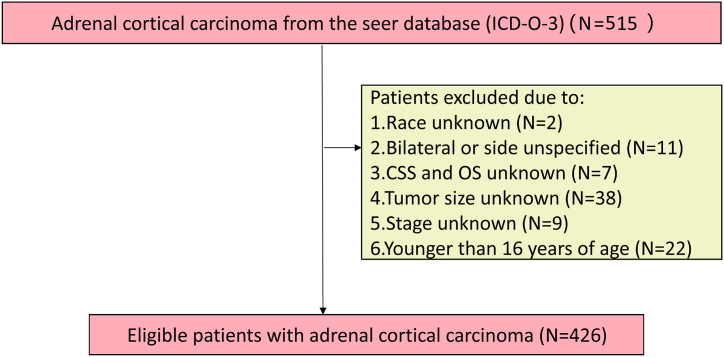
The flow chart of selection.

Patients were diagnosed as adrenal gland (primary site C74.0) with adrenal cortical carcinoma (ICD-0-3 Hist/behav code 8370) between 2004 and 2015 year. Based on the SEER Combined Summary Stage 2000 (2004–2017), the stage of tumor was restaged as localized only (I), regional lymph nodes involved only (II), regional by direct extension only (III), regional by both direct extension and lymph node involvement (IV) and distant site(s)/node(s) involved (V) [24]. The inclusion criteria were as followings: (1) Primary tumor site is adrenal gland. (2) histology is cortical carcinoma. (3) complete clinicopathological and follow-up data. Statistical variables for patients included: sex, race, age, laterality, surgery, tumor size, stage, cause-specific death classification, survival months, radiotherapy (yes, none/unk), chemotherapy (yes, no/unk). The exclusion criteria were as followings: We excluded ACC patients with race unknown, bilateral position and position unknown, surgery unknown, stage unknown, cause-specific death classification unknown and overall survive unknown, tumor size unknown, and patients younger than 16 years of age (Fig. 1).

### 2.3 Cox regression analysis and propensity score matching

In total, 426 participants were enlisted in this study, and the predetermined endpoints were cancer-specific survival (CSS) and overall survival (OS). CSS was defined as the time from the date of diagnosis to the date of death caused by ACC, and OS was defined as the time from the date of diagnosis to the date of caused by any cause. Univariate cox regression models were employed to investigate the risk factors for CSS and OS, and multivariable cox models were utilized to determine the independent risk factors for CSS and OS. Besides, propensity score matching (PSM) was utilized to conduct subgroup analysis for surgery alone and surgery + chemotherapy (CT).

### 2.4 Statistical analysis

We employed the R software, version 4.2.1 (The R Foundation for Statistical Computing, http://www.r-project.org) for statistical analysis. Categorical variables were compared using the Pearson's chi-squared test. The Mann–Whitney *U* test was performed for continuous variables. Univariable and multivariable cox regression analysis were performed to identify the prognostic effect of tumor size and others on CSS and OS, with the hazard ratio (HR) and its 95% confidence interval (Cl). CSS and OS were estimated using the Kaplan–Meier method, and differences were assessed using the log-rank test. The X-tile software version 3.6.1 was utilized to identify the optimal tumor size cutoff value. This choice was informed by X-tile's advanced statistical capabilities, particularly suited for biomarker data analysis. The software excels in applying minimum *P*-value methods for effective data partitioning, enabling precise determination of significant cutoff points within our study. This approach facilitates the identification of meaningful group differences across various sample sets, thereby establishing a scientifically rigorous and accurate benchmark for tumor size assessment. Statistical significance was considered *P*-value < 0.05.

## 3 Results

### 3.1 Patient characteristics

A total 426 adult patients with ACC were identified according to the inclusion and exclusion criteria (Fig. 1). The demographic and clinical characteristics of patients in total cohort are listed in Table 1. The median follow-up time, age and tumor size at diagnosis were 29.0 (11.0–79.5) months, 57 (46–66) years and 10.0 (7.0–15.0) cm, respectively. Among the total cohort, 45.1% of patients (n = 192) were ≤ 55 years old, 41.1% (n = 175) were male, 86.9% (n = 370) were white. As for tumor stage, most was diagnosed as non-metastasis ACC (64.1%, n = 273).

**Table 1:** Characteristics of patients with ACC in total cohort.

Characteristics	*N = 426*	Percent (%)
**Age (year)**	57 (46–66)	–
≤55.0	192	(45.1%)
>55.0	234	(54.9%)
**Sex**		
Male	175	(41.1%)
Female	251	(58.9%)
**Race**		
White	370	(86.9%)
Others	56	(13.1%)
**Laterality**		
Left	214	(50.2%)
Right	212	(49.8%)
**Surgery**		
No	100	(23.5%)
Yes	326	(76.5%)
**Tumor size (cm)**		
Median (IQR)	10.0 (7.0–15.0)	(100%)
**Stage**		
Non-metastasis	273	(64.1%)
Metastasis	153	(35.9%)
**Chemotherapy**		
No	261	(61.3%)
Yes	165	(38.7%)
**Radiotherapy**		
No	360	(84.5%)
Yes	66	(15.5%)

Categoric data are expressed as number (%) and continuous data as median (IQR). Bold values are statistically significant (*P* < 0.05).

### 3.2 Univariable and multivariable cox analysis (interquartile of tumor size) for cancer-specific survival and overall survival

The interquartile range of tumor size was used to divide patients in the total cohort into three groups (<7.0 cm, 7.0–15.0 cm, >15.0 cm). As Supplementary Tables S1 and 2 showed, Univariable and multivariable analysis were conducted, and we found that age, tumor size (Quartile of tumor size), stage and surgery were independently associated with CSS and OS. Kaplan-Meier survival curves indicated that the OS and CSS of ACC patients with larger tumors was were significantly lower than those with smaller tumors (Supplementary Fig. S1A and B), which is consistent with the results of univariable and multivariable cox analysis.

### 3.3 Determination of optimal tumor size cutoff value for ACC patients

**Fig. 2:**
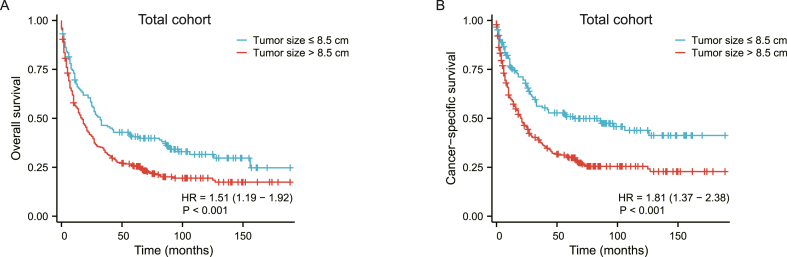
Kaplan–Meier survival curves on overall survival (A) and cancer-specific survival (B) for ACC patients were stratified by tumor size (≤8.5 cm vs > 8.5 cm).

To identify the optimal cutoff value of tumor size that maximized prognosis difference, *X*-tile software was performed based on CSS and OS of total 426 ACC patients and we found the best cutoff value was 8.5 cm (Supplementary Fig. S2A and B). As Table 2 showed, univariable cox analysis indicated that age, surgery treatment, tumor size (cutoff value), tumor stage and chemotherapy were significantly associated with the prognosis of ACC patients for CSS and OS respectively. In the multivariable cox analysis, after adjustment for confounding factors, increased age [HR (95% CI):1.02 (1.01–1.03), *P* < 0.001], larger tumor size [HR (95% CI): 1,81 (1.36–2.39), *P* < 0.001] and metastasis disease [HR (95% CI): 2.80 (2.01–3.91), *P* < 0.001] were independently associated with worse cancer-specific survival (CSS), while receiving surgery treatment [HR (95% CI): 0.29 (0.20–0.41), *P* < 0.001] was associated with better CSS. In the OS analysis, we also observed that surgery [HR (95% CI):0.35 (0.25–0.47), *P* < 0.001] had a favorable correlation with survival and age [HR (95% CI):1.02 (1.01–1.03), *P* < 0.001], metastasis [HR (95% CI):2.54 (1.87–3.45), *P* < 0.001], and larger tumor size [HR (95% CI):1.51 (1.18–1.93), *P* < 0.001] had a negative impact (Table 3). The Kaplan-Meier method and log-rank test demonstrated statistically significant differences in CSS and OS between tumor size ≤ 8.5 cm and > 8.5 cm groups in total cohort (*P* < 0.001) (Fig. 2A and B).

**Table 2:** Univariable and multivariable Cox regression analysis (cutoff value of tumor size) for cancer-specific survival in patients with ACC.

Characteristics	Univariable analysis		Multivariable analysis	
	HR (95% CI)	*P*-Value	HR (95% CI)	*P*-Value
**Age (years)**	1.02 (1.01–1.03)	**< 0.001**	1.02 (1.01–1.03)	**< 0.001**
**Sex**				
Male	ref			
Female	0.95 (0.74–1.21)	0.673		
**Race**				
White	ref			
Others	0.74 (0.50–1.08)	0.115		
**Laterality**				
Left	ref			
Right	1.02 (0.80–1.30)	0.865		
**Surgery**				
No	ref		ref	
Yes	0.17 (0.13–0.23)	**< 0.001**	0.29 (0.20–0.41)	**< 0.001**
**Tumor size (cm)**				
≤8.5 cm	ref		ref	
> 8.5 cm	1.81 (1.37–2.38)	**< 0.001**	1.81 (1.36–2.39)	**<0.001**
**Stage**				
Non-metastasis (I-IV)	ref		ref	
Metastasis (V)	4.32 (3.36–5.57)	**< 0.001**	2.80 (2.01–3.91)	**< 0.001**
**Chemotherapy**				
No	ref		ref	
Yes	1.42 (1.11–1.82)	**0.005**	0.94 (0.71–1.24)	0.665
**Radiotherapy**				
No	ref			
Yes	0.82 (0.57–1.16)	0.257

**Table 3:** Univariable and multivariable Cox regression analysis (cutoff value of tumor size) for overall survival in patients with ACC.

Characteristics	Univariable analysis		Multivariable analysis	
	HR (95% CI)	*P*-Value	HR (95% CI)	*P*-Value
**Age (years)**	1.02 (1.01–1.03)	**< 0.001**	1.02 (1.01–1.03)	**< 0.001**
**Sex**				
Male	ref			
Female	0.91 (0.72–1.13)	0.386		
**Race**				
White	ref			
Others	0.75 (0.53–1.06)	0.099		
**Laterality**				
Left	ref			
Right	1.05 (0.84–1.31)	0.682		
**Surgery**				
No	ref		ref	
Yes	0.20 (0.15–0.26)	**< 0.001**	0.35 (0.25–0.47)	**< 0.001**
**Tumor size (cm)**				
≤8.5 cm	ref		ref	
>8.5 cm	1.51 (1.19–1.92)	**< 0.001**	1.51 (1.18–1.93)	**< 0.001**
**Stage**				
Non-metastasis (I-IV)	ref		ref	
Metastasis (V)	3.68 (2.92–4.64)	**< 0.001**	2.54 (1.87–3.45)	**< 0.001**
**Chemotherapy**				
No	ref		ref	
Yes	1.28 (1.02–1.60)	**0.034**	0.93 (0.72–1.20)	0.559
**Radiotherapy**				
No	ref			
Yes	0.80 (0.58–1.11)	0.186

### 3.4 Impact of tumor size on cancer-specific survival and overall survival in subgroups for ACC patients in total cohort

**Fig. 3:**
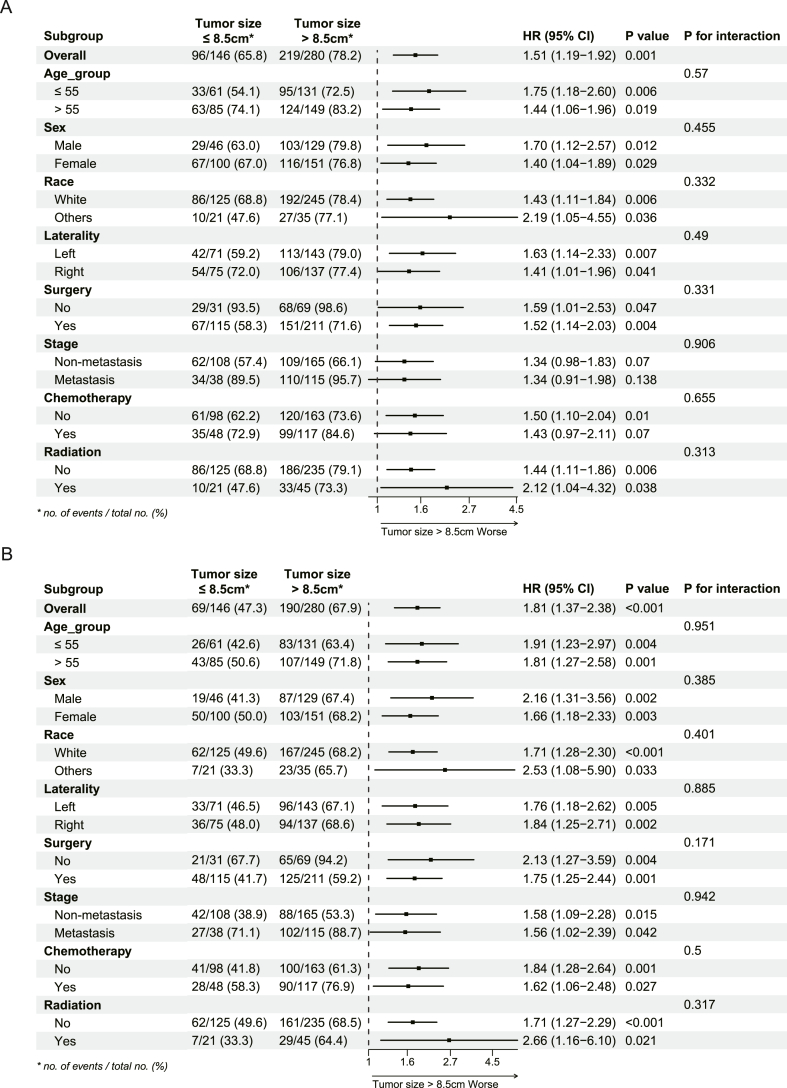
Subgroup analysis for cancer-specific survival and overall survival.

To illustrate the interaction between other risk factors and tumor size, we then performed subgroup analysis in total cohort. Fig. 2A and B indicated that tumor size >8.5 cm was significantly associated with poor prognosis for OS and CSS. However, the forest plot (Fig. 3A and B) for subgroup analysis exhibited a consistent proportional effect of tumor size and lacked statistical significance across all eight pre-specified subgroups (all interaction *P* > 0.05) in OS and CSS analysis.

### 3.5 Effect of tumor size on cancer-specific survival and overall survival in ACC patients with chemotherapy

**Fig. 4:**
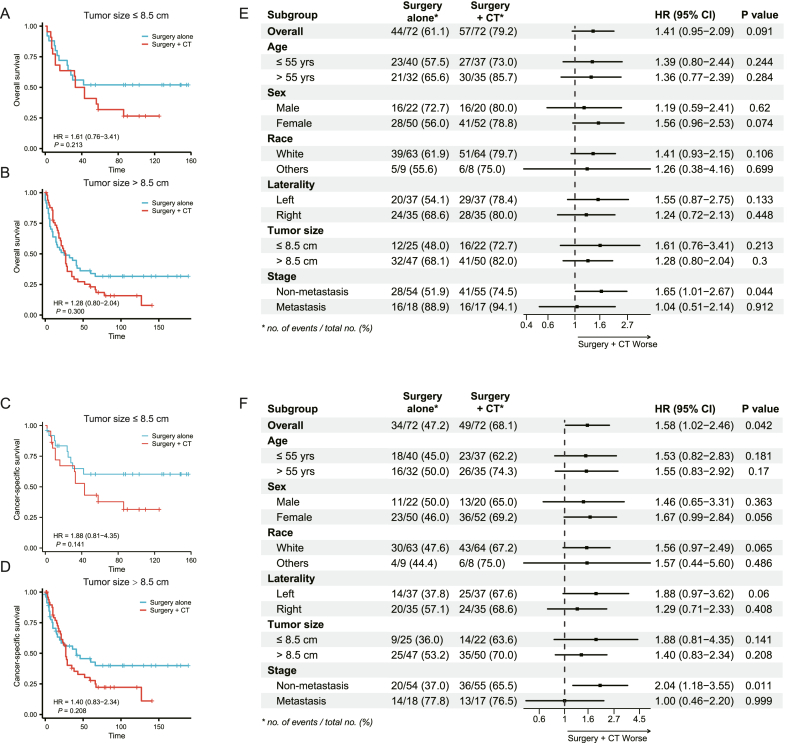
Effect of chemotherapy on overall survival (A.B) and cancer-specific survival (C, D) in ACC patients with tumor size ≤ 8.5 cm (A) and > 8.5 cm (B), and all prespecified groups (E, F) with univariable analysis.

In order to elaborate the effect of tumor size for chemotherapy on CSS and OS in ACC patients, two additional cohorts (surgery alone and surgery + chemotherapy (CT)) were extracted from the total cohort based on whether patients had received surgery and chemotherapy, with patients who received radiotherapy (15.5%, n = 66) excluded. The median follow-up time for surgery alone cohort (N = 188) and surgery + CT cohort (N = 84) were 36.0 (11.0–90.5) and 27.0 (10.0–58.0) months, respectively. 272 patients, who met the inclusion and exclusion criteria, were extracted. Besides, Propensity score matching (PSM) was employed, and all baseline characteristics were well balanced (*P* > 0.05) in Table 4. As shown in Fig. 4A–F, the effect of chemotherapy on OS and CSS in all prespecified subgroups was examined. Interestingly, we found that ACC patients could not benefit from chemotherapy in terms of OS and CSS. Of note, chemotherapy was unable to provide survival benefits no matter in patients with larger tumors (size > 8.5 cm) or those with smaller tumors (≤8.5 cm).

**Table 4:** Characteristics of patients with ACC in different treatment cohort before and after propensity score matching.

Characteristics	Unmatched			Matched		
	Surgery alone cohort N = 188	Surgery + CT cohort N = 84	*P*-value	Surgery alone cohort N = 72	Surgery + CT cohort N = 72	*P*-value
**Age**			0.116			0.955
Median (IQR)	57 (48, 67)	54 (44, 64)		54 (44, 60)	53 (42, 64)	
**Sex**			0.062			0.714
Male	83 (44.1%)	27 (32.1%)		22 (30.6%)	20 (27.8%)	
Female	105 (55.9%)	57 (67.9%)		50 (69.4%)	52 (72.2%)	
**Race**			0.478			0.796
White	162 (86.2%)	75 (89.3%)		63 (87.5%)	64 (88.9%)	
Other	26 (13.8%)	9 (10.7%)		9 (12.5%)	8 (11.1%)	
**Laterality**			0.613			>0.999
Left	98 (52.1%)	41 (48.8%)		37 (51.4%)	37 (51.4%)	
Right	90 (47.9%)	43 (51.2%)		35 (48.6%)	35 (48.6%)	
**Size2**			0.175			0.594
≤8.5 cm	72 (38.3%)	25 (29.8%)		25 (34.7%)	22 (30.6%)	
>8.5 cm	116 (61.7%)	59 (70.2%)		47 (65.3%)	50 (69.4%)	
**Stage**			**<0.001**			0.846
Non-metastasis (I-IV)	166 (88.3%)	56 (66.7%)		54 (75.0%)	55 (76.4%)	
Metastasis (V)	22 (11.7%)	28 (33.3%)		18 (25.0%)	17 (23.6%)

## 4 Discussion

Our study demonstrated that the tumor size of ACC is an independent prognostic factor by univariable cox and multivariable cox regression analysis. Kaplan-Meier survival curves show the prognosis of small tumor size was significantly better than large tumor size. In order to confirm the optimal cutoff value of tumor size, X-tile software was used, and Kaplan-Meier survival indicated that tumor size > 8.5 cm had worse poor prognosis (Fig. 2A and B). Besides, Given the limitations of the available data, it is challenging to conclusively determine the benefit of chemotherapy in adult ACC patients across different tumor size ranges.

Adrenocortical carcinoma (ACC) is an extremely uncommon and malignant condition. Although the diagnosis and treatment of other types of solid malignant tumors have been improved in the past decades, ACC remains a malignancy characterized by high mortality rates and a grim prognosis. The prognosis of ACC is related to many factors. Ayala-Ramirez M et al. reported that older age at diagnosis, functioning tumors, and incomplete resections are clinical factors associated with a less favorable survival [25]. An analysis of data from the National Cancer Database (NCDB) from 1985 to 2005 on 3982 patients with pathologically confirmed ACC implied that an age older than 55 years, large locally invasive tumors, adrenal cortical carcinoma involving marginal and metastatic diseases were associated with shorter survival [26]. In our study of 426 patients with pathologically confirmed ACC, increased age, larger tumor size and metastasis disease were independently associated with worse cancer-specific survival and overall survival. Similarly, Cord S et al. suggested that tumor size is a useful way for predicting the likelihood of ACC [27]. Jonathan J et al. implied that tumor size of ACC ≥ 5 cm increased the margin positivity rate and affected the prognosis of ACC patients [28].

To illustrate the interaction of tumor size with other factors, the forest plot for total cohort was manipulated (Fig. 3A and B), and showed that the proportionate effect of larger tumor size on OS and CSS remained constant across the eight pre-specified subgroups. The impact of tumor size on OS and CSS is apparent and is unlikely to be influenced by the assigned subgroup. Recently research found that race was not an independent factor for survival, but researchers found differences among other factors [29,30]. In our research, the forest plot (Fig. 3A and B) for subgroup analysis exhibited a consistent proportional effect of tumor size and lacked statistical significance across all eight pre-specified subgroups, and suggested that there was no significant difference in tumor size across the race group.

In our study, we have adopted an 8.5 cm cutoff for primary tumor size in adrenocortical carcinoma (ACC), representing a novel approach to prognostic stratification. This decision stands in contrast to the traditional 5 cm threshold used by AJCC and ENSAT systems for distinguishing between stage I and II ACC, which primarily focuses on anatomical extent. While the conventional 5 cm cutoff has been a longstanding measure in ACC staging, our findings suggest that it may not fully capture the prognostic subtleties between lower and higher risk categories of ACC.

Our comprehensive analysis, leveraging the statistical robustness of the “X-Tile” program, has identified 8.5 cm as a more discriminative marker, delineating a clearer demarcation in cancer-specific survival. This threshold indicates that patients with tumors larger than 8.5 cm are likely to experience significantly different clinical outcomes compared to those with smaller tumors. The decision to set the cutoff at 8.5 cm was not arbitrary but rather a result of meticulous statistical evaluation, providing a novel insight into the prognostic stratification of ACC. This finding challenges the current staging paradigm and suggests that the 8.5 cm cutoff may be a more effective tool for stratifying ACC patients into distinct prognostic groups. Such an approach could enhance the precision of treatment decision-making, steering it towards a more personalized and outcome-driven model. It underscores the need for an evolving staging criteria in ACC, one that integrates both anatomical and prognostic data for a more comprehensive understanding of patient outcomes.

Scollo C et al. reported that tumor size is the risk factors significantly associated with recurrence [31]. Hue JJ et al. found that tumors ≥ 5 cm were associated with an increased conversion rate and subsequent increase in margin positivity [28]. According to a recent study, tumor growth correlated inversely with CXCL12 suggesting that local CXCL12 may impair the primary tumor cell response to the ligand gradient that may contribute to driving the tumor progression. CXCL12 negatively correlated with tumor size, this may explain that larger tumors have a worse prognosis [32]. Our study does not negate the importance of established staging systems but suggests that integrating our findings with traditional criteria could yield a more nuanced and effective staging model for ACC. By shifting the focus from purely anatomical considerations to a blend of anatomical and prognostic factors, we advocate for a paradigm shift in ACC management, aiming for a staging system that is as dynamic and multifaceted as the disease itself.

In addition, surgery remains curative treatment for ACC and improves survival, even in metastatic disease [33]. Our results are consistent with previous experiments, and surgery is a curative treatment for ACC. Patients with ACC are recommended for surgery, while larger tumor size has worse prognosis.

The use of chemotherapy for ACC was always controversial. A meta-analysis showed that the administration of adjuvant chemotherapy (mitotane) decreased the risk of mortality [7], Terzolo M et al. performed a retrospective analysis involving 177 patients with adrenocortical cancer who had undergone radical surgery at 8 centers in Italy and 47 centers in Germany between 1985 and 2005. It is concluded that mitotane may prolong recurrence-free survival in patients with radically resected adrenocortical carcinoma [34]. On the contrary the results of ADIUVO trial recently failed to show that administration of mitotane improved relapse free survival and overall survival [35]. A multi-institutional study also demonstrated that delivery of mitotane therapy was not associated with improved patient outcomes [36]. In our research, although receiving chemotherapy is not an independent prognostic factor in the multivariate Cox analysis, it is significantly related to poor prognosis of ACC patients in the univariable cox analysis. For secondary objective of our research to explore the effect of tumor size on CSS and OS in ACC patients with chemotherapy (Table 4), we divided the ACC patients into only surgery group and surgery + CT for subgroup analysis, and propensity score matching was employed. As shown in Fig. 4, Given the limitations of the available data, it is challenging to conclusively determine the benefit of chemotherapy in adult ACC patients across different tumor size ranges. Whether to add chemotherapy is still controversial, we used tumor size as a reference index to evaluate the effect of chemotherapy. Our study demonstrated that tumor size alone seemed unable to identify patients who would benefit from chemotherapy (mitotane). These controversial conclusions, may be attributed to the small sample size due to the rarity of ACC, insufficient blood concentration of chemotherapy drugs [37], potential for selection bias, and lack of distinctive stage-wise design in some studies. In addition, mitotane is usually used in combination with other cytotoxic chemotherapy drugs, so it is impossible to distinguish whether the tumor response represents mitotane, chemotherapy drugs or their combined effects. Therefore, few effective therapeutic experiments are currently available. The impacts of tumor size in combination with chemotherapy on CSS and OS urges to be further analyzed in the future.

Potential limitations should be considered. Specifically, we acknowledge that the SEER database, while extensive, may not capture all relevant clinical variables that could influence the outcomes of patients with adrenocortical carcinoma (ACC). These variables include, but are not limited to, detailed treatment regimens, comorbid conditions, and certain biochemical markers that might impact the prognosis of ACC. Additionally, the lack of information on genetic and molecular characteristics of the tumors in the SEER database is a notable limitation, as these factors are increasingly recognized as important in the pathogenesis and progression of ACC. Furthermore, we recognize that the retrospective nature of the study and the reliance on registry data might introduce selection bias and limit the generalizability of our findings. The data reflects treatment and outcomes in a real-world setting, which, while valuable, might not strictly represent the outcomes of controlled clinical trials.

## 5 Conclusion

We found tumor size (8.5 cm) is an independent factor in adrenocortical carcinoma and tumor size >8.5 cm was significantly associated with poor prognosis. Given the limitations of the available data, it is challenging to conclusively determine the benefit of chemotherapy in adult ACC patients across different tumor size ranges. Whether to add chemotherapy after surgery is still controversial. We propose that the tumor size of ACC may be interesting factor for further analysis, such as the choice of chemotherapy or radiotherapy. Therefore, we suggest incorporating this value into the risk classification system to enhance the accuracy of ACC prognostic prediction.

## Ethics approval and ethics approval

Approval from the ethical board for this study was not required because of the public nature of all the data. This study did not involve personal identifying information or interact with human individuals, and informed consent was not required.

## Funding

This work was supported by the National Natural Science Foundation of China（82203720, 82203188, 82002682, 81972731, 81773026, 81972383), the 10.13039/501100012245Science and Technology Planning Project of Guangdong Province (2020A1515111119, 2019A1515010188) and the Guangzhou Municipal Basic Research Program Jointly Funded by City, University, and Enterprise Special Project (2024A03J0907).

## Data availability statement

To receive the relevant data, it is possible to make reasonable inquiries by getting in touch with the corresponding authors.

## CRediT authorship contribution statement

**Yi Huang:** Writing – original draft, Supervision, Software, Resources, Methodology, Investigation. **Libo Liu:** Writing – original draft, Supervision, Software, Methodology. **Qinghua Gan:** Software, Data curation, Conceptualization. **Zefeng Shen:** Resources, Investigation, Formal analysis. **Yuhui Yao:** Resources, Project administration. **Chengxiao Liao:** Methodology, Investigation. **Sihong Lu:** Software, Investigation. **Yitong zou:** Visualization, Conceptualization. **Yaqiang Huang:** Visualization, Validation, Supervision, Software. **Jianqiu Kong:** Writing – review & editing, Writing – original draft, Visualization, Supervision, Software, Conceptualization. **Xinxiang Fan:** Writing – review & editing, Writing – original draft, Visualization, Validation, Supervision, Formal analysis.

## Declaration of competing interest

The authors declare that they have no known competing financial interests or personal relationships that could have appeared to influence the work reported in this paper.
