# Supplementary figures and images for: Estimation of the tumor size at cure threshold among adult patients with adrenocortical carcinoma: A populational-based study
Source: Heliyon. 2024 Mar 22;10(7):e28160. doi: 10.1016/j.heliyon.2024.e28160 (PMC10987901; doi:10.1016/j.heliyon.2024.e28160)

A

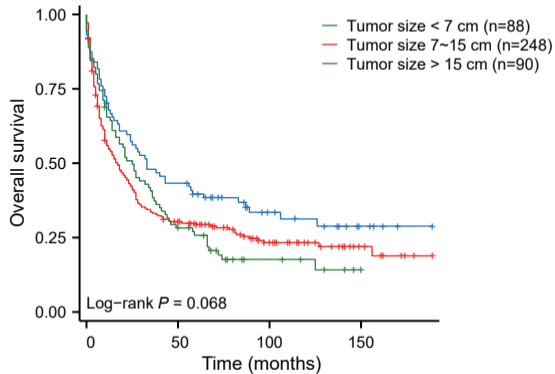

B

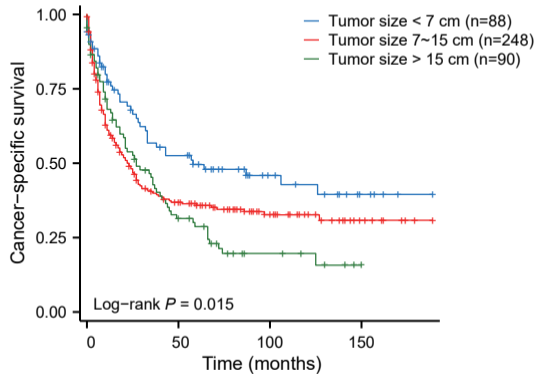

Supplement: Multimedia component 1 [file mmc1.pdf]

A

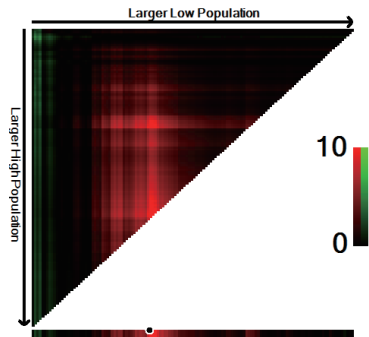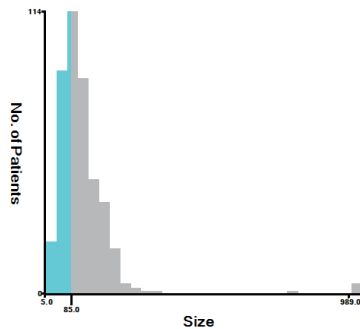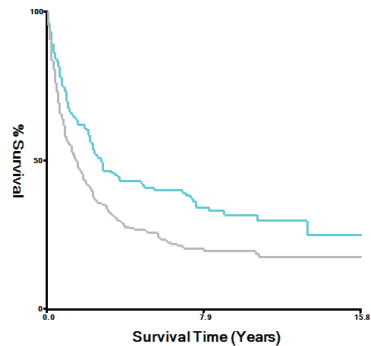

B

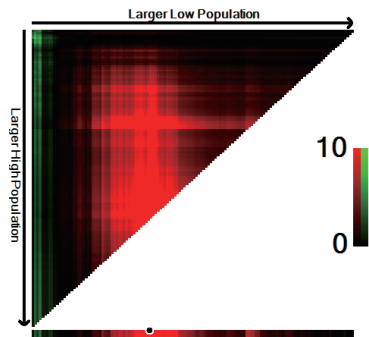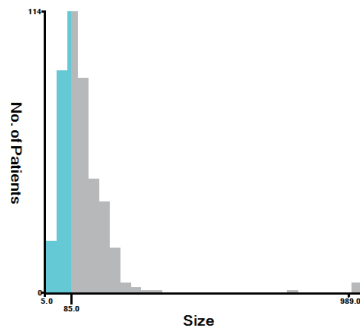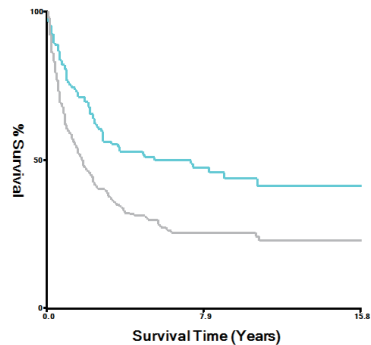

Supplement: Multimedia component 2 [file mmc2.pdf]
