# Supplementary material for: Estimation of the tumor size at cure threshold among adult patients with adrenocortical carcinoma: A populational-based study
Source: Heliyon. 2024 Mar 22;10(7):e28160. doi: 10.1016/j.heliyon.2024.e28160 (PMC10987901; doi:10.1016/j.heliyon.2024.e28160)
Supplement: Multimedia component 3 [file mmc3.docx]

**Supplement figure legends**

**Supplement figure 1:** Kaplan–Meier survival curves on overall survival (A) and cancer-specific survival (B) for ACC patients were stratified by tumor size (＜7.0 cm,

7-15.0 cm,＞15.0 cm)

**Supplement figure 2:** X-tile software was employed to determine the optimal cut off value of tumor size for OS (A) and CSS (B)

**Supplement Table 1. Univariable and multivariable Cox regression analysis for cancer-specific survival in patients with ACC.**

| **Characteristics** | **Univariable analysis** | | | **Multivariable analysis** | |
| --- | --- | --- | --- | --- | --- |
|  | **HR (95% CI)** | ***P*-Value** |  | **HR (95% CI)** | ***P*-Value** |
| **Age (years)** | 1.18 (1.01-1.03) | **< 0.001** |  | 1.02 (1.01-1.03) | **< 0.001** |
| **Sex** |  |  |  |  |  |
| Male | ref |  |  |  |  |
| Female | 0.95 (0.74-1.21) | 0.673 |  |  |  |
| **Race** |  |  |  |  |  |
| White | ref |  |  |  |  |
| Others | 0.74 (0.50-1.08) | 0.115 |  |  |  |
| **Laterality** |  |  |  |  |  |
| Left | ref |  |  |  |  |
| Right | 1.02 (0.80-1.30) | 0.865 |  |  |  |
| **Surgery** |  |  |  |  |  |
| No | ref |  |  | ref |  |
| Yes | 0.17 (0.13-0.23) | **< 0.001** |  | 0.29 (0.20-0.41) | **< 0.001** |
| **Tumor size (cm)** |  |  |  |  |  |
| ＜7.0 | ref |  |  | ref |  |
| 7-15.0 | 1.48 (1.05-2.09) | **0.025** |  | 1.74 (1.23-2.47) | **0.002** |
| ＞15.0 | 1.78 (1.23-2.58) | **0.002** |  | 2.15 (1.45-3.17) | **<0.001** |
| **Stage** |  |  |  |  |  |
| Non-metastasis (I-IV) | ref |  |  | ref |  |
| Metastasis (V) | 4.23 (3.36-5.57) | **< 0.001** |  | 2.73 (1.94-3.83) | **< 0.001** |
| **Chemotherapy** |  |  |  |  |  |
| No | ref |  |  | ref |  |
| Yes | 1.42 (1.11-1.82) | **0.005** |  | 0.97 (0.74-1.29) | 0.852 |
| **Radiotherapy** |  |  |  |  |  |
| No | ref |  |  |  |  |
| Yes | 0.82 (0.57-1.16) | 0.257 |  |  |  |

**Supplement Table 2. Univariable and multivariable Cox regression analysis for overall survival in patients with ACC.**

| **Characteristics** | **Univariable analysis** | | | **Multivariable analysis** | |
| --- | --- | --- | --- | --- | --- |
|  | **HR (95% CI)** | ***P*-Value** |  | **HR (95% CI)** | ***P*-Value** |
| **Age (years)** | 1.02 (1.01-1.03) | **< 0.001** |  | 1.02 (1.01-1.03) | **< 0.001** |
| **Sex** |  |  |  |  |  |
| Male | ref |  |  |  |  |
| Female | 0.91 (0.72-1.13) | 0.386 |  |  |  |
| **Race** |  |  |  |  |  |
| White | ref |  |  |  |  |
| Others | 0.75 (0.53-1.06) | 0.099 |  | 0.75 (0.53-1.06) | 0.098 |
| **Laterality** |  |  |  |  |  |
| Left | ref |  |  |  |  |
| Right | 1.05(0.84-1.31) | 0.682 |  |  |  |
| **Surgery** |  |  |  |  |  |
| No | ref |  |  | ref |  |
| Yes | 0.20 (0.15-0.26) | **< 0.001** |  | 0.32 (0.23-0.45) | **< 0.001** |
| **Tumor size (cm)** |  |  |  |  |  |
| ＜7.0 | ref |  |  | ref |  |
| 7-15.0 | 1.35 (1.00-1.83) | **0.047** |  | 1.58 (1.17-2.14) | **0.003** |
| ＞15.0 | 1.49 (1.07-2.08) | **0.018** |  | 1.77 (1.25-2.51) | **0.001** |
| **Stage** |  |  |  |  |  |
| Non-metastasis (I-IV) | ref |  |  | ref |  |
| Metastasis (V) | 3.68 (2.92-4.64) | **< 0.001** |  | 2.55 (1.87-3.47) | **< 0.001** |
| **Chemotherapy** |  |  |  |  |  |
| No | ref |  |  | ref |  |
| Yes | 1.28 (1.02-1.60) | **0.034** |  | 0.94 (0.73-1.22) | 0.652 |
| **Radiotherapy** |  |  |  |  |  |
| No | ref |  |  |  |  |
| Yes | 0.80 (0.58-1.11) | 0.186 |  |  |  |
